# Executive functioning in adolescents with internalizing disorders: a systematic review

**DOI:** 10.1007/s00787-025-02826-2

**Published:** 2025-10-13

**Authors:** Géraldine P. Fontaine, Kimberly V. Blake, Nastassja Koen, Dan J. Stein, Åsa Hammar, Nynke A. Groenewold

**Affiliations:** 1 Neuroscience Institute, Department of Psychiatry and Mental Health, University of Cape Town, Cape Town, South Africa; 2 South African Medical Research Council (SAMRC) Unit on Risk and Resilience in Mental Disorders, Cape Town, South Africa; 3 Department of Biological and Medical Psychology, University of Bergen, Bergen, Norway; 4 Department of Clinical Sciences Lund, Psychiatry, Faculty of Medicine, Lund University, Lund, Sweden; 5 Department of Psychiatry, Skåne University Hospital, Lund, Sweden

**Keywords:** Neuropsychology, Executive functioning, Internalizing disorders, Adolescents

## Abstract

Internalizing disorders (INTs), including anxiety (AD) and depressive disorders (DD), frequently emerge during adolescence. Studies suggest that certain core domains of executive functioning (EF), i.e. inhibition, shifting and working memory (WM) may show selectively lower performance in certain INTs. This systematic review aimed to evaluate the evidence of associations between INTs and EF in adolescents. A systematic search was conducted in Medline, Psych-INFO, Scopus, and Web of Science in May 2023. Inclusion focused on adolescents (12–17) with AD (including obsessive-compulsive disorder (OCD) and post-traumatic stress disorder (PTSD)) or DD. After screening 2,551 titles/abstracts, 818 records underwent full-text review, on which independent reviewers reached 93% agreement on eligibility. EF parameters, measured through task-performance or ratings, were extracted from 32 eligible articles published since 2014. Performance-based EF differences were reported in 22 studies, most frequently in adolescents with DD. Task-based inhibition showed lower scores most consistently in DD (*n* = 5). Findings were mixed across other INTs. Three studies found evidence for improved inhibition performance in OCD (*n* = 2) and AD (*n* = 1). Finally, 6 studies reporting on self- or parent-rated EF found significant difficulties across EF domains, of which 3 contrasted with intact performances on task-based measurements of EF. Findings suggest both objective and subjective EF difficulties across INTs with no conclusive evidence for selective domain-specific differences. We highlight a discrepancy between subjectively experienced or observed deficits in daily EF and performance on structured tasks. This suggests that rating-based EF may be more sensitive for capturing subtle EF difficulties and therefore adds value in research and clinical settings. Finally, the quality of studies is discussed and directions for future studies are identified, namely, the use of both task and rating-based measures of EF as well as inclusion of larger sample sizes.

## Introduction

Executive functions (EF) are defined as a collection of higher-order cognitive processes that coordinate and regulate lower-level processes to enable purposeful, goal-directed behavior [1]. Performance difficulties in these processes are associated with most forms of psychopathology [2], including internalizing disorders (INTs). Overall, these processes, which are refined during the protracted maturation of the prefrontal cortex in adolescence [3], play a central role in academic performance, as well as emotional and social maturation of adolescents [4, 5]. INTs, encompassing anxiety disorders (AD), depressive disorders (DD), obsessive-compulsive disorder (OCD) and post-traumatic stress disorder (PTSD), are highly prevalent mental health conditions characterized by the tendency to direct distress inwards [6]. INTs frequently co-occur, and typically emerge during adolescence [7–9]. There may be bidirectional relationships between EF and INT during adolescence [10]; INTs that emerge during this vulnerable period may interfere with the healthy development of EF abilities [11] and EF difficulties may disrupt emotional regulation and worsen INTs [12, 13].

EF can be viewed through two complementary lenses: as a unified system and as distinct components. Support for unity comes from evidence of a superordinate cognitive control network that coordinates various EF [14]. Nevertheless, several findings suggest these functions are also separable: different brain regions are recruited for different executive tasks, particularly in prefrontal, cingulate, and subcortical areas [15], and performance on different types of EF tasks only show modest correlations [16]. Consequently, the unity and diversity model [1] has gained traction. Although definitions of EF continue to evolve and are not always fully convergent across the literature, this review focuses on three core EF domains—inhibition, shifting, and working memory—based on Miyake et al.‘s [1, 16, 17] influential unity and diversity model, which coincides with Diamond’s [18] more recent conceptualization of EF. Miyake’s model proposes that EF share common underlying processes while maintaining distinct components, reconciling evidence for both general and specific aspects of executive control. Based on confirmatory factor analysis of several EF tasks, this model identifies a taxonomy proposing these three “core”, separable, EF domains: inhibition (i.e., suppressing or controlling attention, thoughts, behaviors), shifting (i.e., shifting flexibly between tasks/sets), and working memory (i.e., maintaining/manipulating data not perceptually present) [1, 16, 18].

A meta-analysis by Wagner et al. [19] revealed that children and adolescents with major depressive disorder (MDD) exhibited significantly worse EF task performance compared to healthy controls (HC), with the most substantial differences observed in inhibition. These findings were further supported by Kavanaugh et al. [20], who demonstrated that among the EF subdomains, lower performance in response inhibition displayed the strongest association with a clinical diagnosis of MDD. Castaneda et al. [21] conducted a review in young adults with anxiety and/or depressive disorders and found that the presence of EF difficulties in MDD were evident across multiple domains. On the other hand, EF difficulties in AD varied depending on the specific anxiety subtype, with only OCD participants exhibiting significantly lower EF performance compared to other AD - particularly in set shifting, working memory and verbal fluency. Furthermore, a meta-analysis [5] in children and adolescents with post-traumatic stress symptoms (PTSS) showed overall poorer EF compared to HCs and trauma-exposed controls without PTSS, with no clear evidence for specific subdomains having a more reduced performance than others. Importantly, findings by Mullin et al. [4] demonstrated robust links between more severe anxiety and depressive symptoms and lower self- and parent-reported EF abilities in adolescents, as measured by the Behavior Rating Inventory of Executive Function (BRIEF) [22]. These studies suggest that subjective ratings of EF difficulties are closely tied to the expression of INTs in adolescents, highlighting the transdiagnostic relevance of EF in this population.

However, the majority of studies on EF in INTs have focused on adult populations, overlooking the crucial developmental period of adolescence – which is critical for the progression of EF skills and its susceptibility to emergence of INTs. While numerous reviews have shown strong evidence of lower performance on EF tasks in adults with INTs [2, 23–26], reviews in adolescents have been less conclusive [27, 28]. Existing reviews have often used broad age ranges, combining children and adolescents from 6 to 19 years [19, 28] or adolescents and young adults from 12 to 25 years [29], potentially obscuring age-specific findings. Our focus on the adolescent period (12–17 years) addresses this gap by targeting a critical neurodevelopmental window characterized by significant changes in brain regions supporting EF, particularly the prefrontal cortex [30, 31]. Importantly, adolescence coincides with a marked increase in the incidence of internalizing disorders [7], with approximately 50% of all lifetime mental disorders having their onset by age 14 [32]. The unique co-occurrence of rapidly developing EFs and emerging psychopathology during adolescence makes this a particularly important developmental stage to examine the relationship between EF and INTs. To the best of our knowledge, no previous review has comprehensively and systematically examined EF performance on both task-based and self-report measures, across a wide spectrum of INTs, in adolescents.

Examining INTs collectively offers methodological and theoretical advantages. These disorders share core phenomenological features, including negative affect, withdrawal behaviors, and emotion dysregulation [33, 34]. INTs are highly co-morbid, with up to 50–70% of adolescents meeting criteria for multiple internalizing diagnoses [35, 36]. Additionally, transdiagnostic research suggests common cognitive and neurobiological mechanisms across these disorders including potential shared executive dysfunction [37, 38]. By examining these disorders together while also analyzing disorder-specific profiles, we can identify both transdiagnostic EF difficulties that may represent broader vulnerability factors and disorder-specific EF patterns that may contribute to unique symptom presentations.

To further our understanding of the nature and extent of EF performance in this population, and to inform theoretical models as well as clinical assessment of internalizing psychopathology in adolescents, we aimed to systematically review recent literature on EF in adolescents with INTs. We specifically aimed to review the literature published between January 2014 and May 2023, on three task-based and self- or parent-rated EF domains (inhibition, shifting and working memory), in adolescents with DD, AD, OCD or PTSD (Tables 1, 2, 3, 4 and 5).

**Table 1:** Executive functioning in adolescents with depressive disorders

Study nr	Paper	Status	Mean age (SD)	Sample size	Diagnostic criteria	Psychiatric co-occurrences	Psychotropic medications	EF domain	Instrument	Results	Statistic
1	*Baller (2021)*	MDD (lifetime)	MDD:16.87 (2.20)	712	Interview *GOASSESS; DSM-IV-TR*	MDD: NR HC: No	MDD: 30% of the sample HC: 13% (Controlled for)	SHIFT	PCET (CNB)	**MDD Subtype 2 < HC (accuracy & speed)** **MDD Subtype 1 > Subtype 2**	***d*** **= .50** ***d*** **= .76**
			HC:16.49 (2.84)	712				WM	Letter N-Back (CNB)	**MDD Subtype 2 < HC (accuracy & speed)** **MDD Subtype 1 > Subtype 2**	***d*** **= .50** ***d*** **= .54**
2	*Chen (2023)*	MDD (current)	MDD:17.07 (0.80)	29	Interview *MDD: DSM-5; GCI-S* ≤ *3* *HC: MINI-Kid*	*NR*	Yes: % NR	SHIFT	WCST	*NS*	*N/A*
			HC:17 (1.20)	22				WM	N-Back	**MDD < HC (mean time)**	***p*** ** = .016**
3	*Dell’Acqua (2023;a)*	MDD (current & lifetime)	MDD: 13.6 (1.09)	29	Interview *K-SADS-PL; DSM-IV*	MDD: OCD (10%), AD (> 50%)	*NR*	INH	Flanker Task	**MDD < HC (speed)**	***p*** ** = .020**
			HC: 16.2 (2.91)	34		HC: No					
4	*Diler (2014)*	MDD (current)	MDD: 15.9 (1.10)	10	Interview *K-SADS-PL; DSM-IV-TR)*	MDD: AD (60%), ADHD (20%)	MDD: 60%	INH	Go/NoGo	*NS (reaction times for NoGo responses)*	*p* = *.56*
			HC:15.6 (1.2)	10		HC: NR	HC: None				
5	*Fisk (2019)*	Elevated depressive symptoms (current)	Elevated: 14.84 (1.36)	29	Other *MFQ* ≥ *27*	*NR*	*NR*	INH	The HSC	*NS (number of errors)* *NS (reaction time)*	*p* = *. 51* *p* = *.06*
			HC: 14.65 (1.52)	29				WM	The KTT	**DEP < HC**	***p*** ** = .001**
6	*Gillespie and Rao (2022)*	MDD	MDD: 16.28 (1.40)	63	Interview *K-SADS-COMP V2.0; DSM-IV*	*NR*	No	GEC	BRIEF-SR	**MDD < HC**	***p*** ** < .01**
			HC: 15.60 (1.45)	75				INH	CWIT (D-KEFS)	*NS*	*N/A*
7	*Hanna (2018)*	MDD (current & lifetime)	MDD: 16.8 (1.4)	36	Interview *K-SADS; DSM-5*	MDD: Yes (% NR) HC: No	MDD: 30% HC: None	INH	Flanker Task	*NS*	*N/A*
			HC: 16.2 (1.8)	89							
8	*Liang (2021)*	MDD (Current & lifetime)	MDD: 17.9 (2.7)	50	Interview *ICD-10*	Yes (% NR)	MDD: Yes (% NR)	WM	MCCB—WMS-III SST	*NS*	*p* = *.576*
			HC:17.7 (3.2)	51			HC: NR				
9	*Onat (2019)*	MDD (Current & lifetime)	MDD-SA: 15.85 (1.02)	32	Interview *K-SADS-PL; DSM-IV*	No	MDD: Yes (% NR)	SHIFT	WCST	*NS*	*N/A*
			MDD w/o SA: 15.9 (1.23)	30			HC: None	INH	Stroop	**Part 1: MDD-SA > HC (time)**	***p*** ** = .029**
			HC: 15.99 (1.37)	30						**Part 3: MDD w/o SA > HC, MDD w/o SA > MDD-SA (correction)**	***p*** ** = .17**
										**Part 4: MDD-SA > HC (time)**	***p*** ** = .015**
10	*Pan (2020)*	MDD (Current; First-episode)	MDD: 16	28	Interview *SCID; DSM-IV*	No	No	INH	SCWT	**Part B: MDD < HC** **Part C: MDD < HC**	***p*** **< .05** ***p*** **< .05**
			HC: 16	24				SHIFT	WCST; TMT-B	*NS*^+^ *(perseverative errors)*	*p* = *.219*
11	*Peters (2019)*	Mixed DD	MDD-CT: 14.45 (1.54)	22	Interview *K-SADS-PL*; *DSM-IV*	MDD-CT: AD (> 50%), PTSD (18.2%), ADHD (22.7%) MDD w/o CT: AD (> 50%), PTSD (5.6%), ADHD (5.6%)	MDD-CT: No MDD w/o CT: 11%	Parent-rated INH	BRIEF-PR	**MDD-CT < HC** **MDD < HC**	***d*** **= .62;** ***p*** **< .05** ***d*** **=** ***.64***
			MDD: 14.61 (1.46)	18		HC: No	HC: No	INH	Go/NoGo	**MDD-CT < HC (accuracy)**	***d*** ** = .54; ** ***p*** ** < .05**
			HC:14.67 (1.37)	30							
12	*Shebab (2016)*	MDD (current)	MDD: 14.83 (1.63)	24	Interview *DAWBA; DSM-IV & ICD-10; CDR-RS* ≥ *40*	MDD: AD (50%), ADHD (8.3%)	MDD: 8.3%	INH	RVP B’’ (CANTAB)	**MDD < HC**	***p*** ** < .05**
			HC: 14.25 (1.59)	24		HC: No	HC: No				
13	*Zhang (2022)*	MDD (current)	MDD-NSSI:16.13 (1.95)	40	Interview *DSM-5*	NR	No	SHIFT	TMT-B	**MDD-NSSI < HC** **MDD < HC** **MDD < HC**	***p*** **< .01** ***p*** **< .01** ***p*** **< .05**
			MDD: 16.67 (1.9)	46					WCST	**MDD-NSSI < HC** **MDD < HC**	***p*** **< .01** ***p*** **< .01**
			HC: 17.07 (3.1)	28							

*SD* Standard deviation, *NR* Not reported, ^+^ after correcting for multiple comparisons, *GCI-S* Clinical Global Impression-Severity scale, *MFQ* The Mood and Feelings Questionnaire, *MDD* Major Depressive Disorder, *MDD-SA *MDD with suicide attempt, *MDD-CT* MDD with childhood trauma, *MDD-NSSI* MDD with non-suicidal self-injury, *HCs* Healthy controls, *WM* Working memory

*PCET* Penn Conditional Exclusion Test, *FT* Flanker Task, *MCCB* The MATRICS Consensus Cognitive Battery, *HSC* The Hayling Sentence Completion task, *KTT* The Keep Track Task, *RPV* Rapid Visual Processing, *GEC* Global Executive Composite score, *DST* Digit Symbol Test, *SCWT* Stroop Color and Word Test

**Table 2:** Executive functioning in adolescents with anxiety disorders

Study nr	Paper	Status	Mean age (SD)	Sample size	Diagnostic criteria	Psychiatric co-occurrences	Psychotropic medications	EF domain	Task	Results	Statistic
14	*Baumel (2022)*	GAD (Current)	GAD: 14.8 (1.6)	50	Interview *PARS* ≥ *15*	GAD: AD (% NR)	None	INH	BRIEF-SR	**GAD < N**	***p*** ** = ** ***.002***
			*HCs matched on age & sex*			N: None		SHIFT		**GAD < N**	***p*** ** < ** ***.001***
								WM		**GAD < N**	***p*** ** < ** ***.001***
15	*Cardinale (2019)*	AD—Not specified (Lifetime & current)	AD: 12.37 (3.03)	35	Interview *K-SADS-PL; DSM-IV*	None	None	INH	Adapted Antisaccade Task	**AD > HC (faster latency to correct antisaccades)**	***p*** ** = ** ***.03***
			HC: 13.09 (3.31)	22							
16	*Jarros (2017)*	AD: SAD, GAD, SeAD, PD	AD-M: 13.0 (2.19)	28	Interview *K-SADS -PL; DSM-IV*	**AD-M:** ADHD (14.3%), MDD (3.6%), SP (32.1%) **AD-S:** ADHD (38.5%), MDD (15.4%), PTSD (7.7%), SP (53.8%) **HC:** ADHD (33.3%), SP (14.8%)	None	INH	Go/NoGo	*NS (total hits)*	*p* = *.583*
			AD-S: 12.2 (1.21)	13				SHIFT	TMT-B, WCST	*NS (TMT-B time)* *NS (TMT-B errors)* *NS (WCST total errors)* *NS (WCST perseverative errors)*	*p* = *.548* *p* = *.662* *p* = *.655* *p* = *.489*
			HC: 12.6 (1.96)	27				WM	Digit Span	*NS (forward)* **AD-M > AD-S = HCs (backward)**	*p* = *.271* ***p*** **=** ***.015***
17	*Kim (2019)*	GAD	GAD: 11.9 (2.9)	34	Interview *K-SADS-PL; DSM-IV*	GAD: PD (11.8%), SAD (17.6%)	GAD: 45%	SHIFT	IDED	**GAD < HC (Simple reversal errors)** GAD = HC (Total reversal errors)	***d*** **=** ***.53; p*** **=** ***.01*** *d* = *31; p* = *.21*
			HC: 13 (2.9)	65		HC: None	HC: None	WM	Spatial Span task	*NS*	*N/A*

*N* Normative sample, *AD* Anxiety disorders, *SeAD* Separation anxiety disorder, *PARS* Pediatric Anxiety Rating Scale, *AD-M* Mild (CGI ≤ 4) anxiety symptoms, *AD-S* Severe (CGI >4) anxiety symptoms, *SWM* Spatial Working Memory from the Comprehensive Attention Test (CAT) battery, *FT* Flanker Task from the CAT, *IDED* Intra–Extra Dimensional Set Shift

**Table 3:** Executive functioning in adolescents with obsessive-compulsive disorder

Study nr	Paper	Status	Mean age (SD)	Sample size	Diagnostic criteria	Psychiatric co-occurrences	Psychotropic medications	EF domain	Task	*Results*	*Statistic*
18	*Bohon (2020)*	*OCD*	OCD: 15.64 (2.01)	11	Interview *K-SADS; SCID*	OCD: AD (45%)	NR	SHIFT	WCST	**OCD < HC (perseverative errors)**	***d*** ** = .81; ** ***p*** ** < .05**
			HC: 15.29 (1.65)	24		HC: No					
19	*Dell’Acqua (2023;b)*	*OCD*	OCD: 12.6 (1.03)	27	Interview *K-SADS-PL*	No	Yes (% NR)	INH	Flanker Task	**OCD > HC (speed in error trials)**	***p*** ** = .006**
			HC: 12.3 (1.10)	27							
20	*Gottwald (2018)*	*OCD*	OCD: 16.6 (1.9)	36	Interview *MINI-Kids*	OCD: No but high scores on BAI & BDI	OCD: 64%	SHIFT	IDED (CANTAB)	*NS*	*N/A*
			HC: 16.6 (2.1)	36		HC: No	HC: no				
7	*Hanna (2018)*	*OCD* *(Lifetime)*	OCD: 15.6 (1.4)	39	Interview *K-SADS-PL;DSM-5*	OCD: Yes (% NR)	OCD: Yes 36 of overall sample (i.e., OCD + MDD) take SSRIs	INH	Flanker Task	**OCD < HC**	***p*** ** < .001**
			HC: 16.2 (1.7)	89		HC: No				**OCD < MDD**	***p*** ** < .01**
21	*Hybel (2017)*	*OCD*	OCD: 13.08 (2.23)	50	Interview *DSM-IV; CY-BOCS* ≥ *16*	OCD: AD (20%)	No	INH	Flanker Task; Stop Signal Task	*NS*	*N/A*
			HC: 13.12 (2.38)	50				SHIFT	IDED; TMT-B	*NS*	*N/A*
								WM	SWM; Spatial Span task	*NS*	*N/A*
								Parent-rated INH SHIFT WM	BRIEF-PR	**OCD < HC**	***d*** ** = .58 – 1.45**
22	*KamberoğluTuran (2023)*	*SPD (OCD)*	SPD:13.9 (1.8)	96	Interview *K-SADS; DSM-5*	OCD: MDD (4.17%), ADHD (12.50%), AD (5.21%), OCD (11.46%)	SPD: NR	INH	BRIEF-PR	**SPD < HC**	***p*** ** = .030**
			HC: 13.4 (1.7)	90			HC: No	SHIFT		*NS*	*p* = *.113*
								WM		*NS*	*p* = *.111*
17	*Kim (2019)*	*OCD*	OCD: 12.6 (2.5)	28	Interview *K-SADS*	OCD: AD (57%), ADHD (10.7%)	OCD: 64%	SHIFT	IDED	*NS*	*N/A*
			HC: 13 (2.9)	65		HC: No	HC: No	WM	Spatial Span Task	*NS*	*N/A*
23	*Marzuki (2021)*	*OCD*	OCD: 15.83	27	Interview *MINI; MINI-Kid*	No	OCD: 59.25%	SHIFT	WCST	*NS (perseverative errors)* *NS (RT)*	*p* = *.54* *p* = *.09*
			HC: 17.17	46			HC: No				
24	*Negreiros (2020; a)*	*OCD*	OCD: 13.38 (2.71)	87	Interview *DSM-IV; ADIS-P*	OCD: AD (21.16%), ADHD (7%), TTM (1%), SPD (1%)	OCD: 26%	INH	Stop Signal Task	*NS*	*N/A*
			HC: 12.18 (3.33)	67		HC: No	HC: 6%	SHIFT	IDED	*NS*	*N/A*
								WM	Spatial Working Memory Task	*NS*	*N/A*
25	*Negreiros (2020;b)*	*OCD*	OCD: 12.82 (2.57)	75	Interview *DSM-IV; ADIS-P*	OCD: AD (45.33%), ADHD (8%),	OCD: 28%	INH	Stop Signal Task	*NS*	*d* = .*21; p* = *.2*
			HC: 12.48 (3.03)	43		SPD (3%)	HC: None	SHIFT	IDED	*NS*	*d* = *.04; p* = *.88*
						HC: No		WM	Spatial Working Memory Task	**OCD < HC**	***d*** ** = ** ***.62; p*** ** = ** ***.003***
								INH SHIFT WM	BRIEF-PR	**OCD < HC** **OCD < HC** **OCD < HC**	***d*** **=** ***.78; p*** **<** ***.001*** ***d*** **=** ***1.36; p*** **<** ***.001*** ***d*** **=** ***.92; p*** **<** ***.001***
26	*Peris (2021)*	*OCD (current)*	TTM: 14.1 (2.1)	22	Interview *DSM-IV; ADIS-IV*	TTM: AD (59%), MDD (18%), ADHD 13,63% OCD: AD (36.36%), MDD (18%), ADHD (4.54%)	No	INH	Modified Flanker Task	*NS*	*p* = *.08*
			OCD: 13.6 (2.4)	22		HC: No					
			HC: 13.2 (2.1)	19							
27	*Pop-Jordanova (2019)*	*OCD*	OCD: 14,5 (2,2)	20	Interview *DSM-IV; K-SADS*	NR (high scores on CBCL)	NR	INH	SCWT	**OCD < HC (interference)**	***p*** ** < ** ***.001***
			HC: NR	20				FLEX	Go/NoGo (VCPT)	**OCD < HC (commission errors)** **OCD < HC (RT variability)**	***p*** **<** ***.01*** ***p*** **<** ***.01***
									WCST	**OCD < HC (perseverative errors)**	***p*** ** < ** ***.001***
28	*Wolff (2019)*	*OCD*	OCD: 13.8 (2.34)	27	Interview *ICD-10; ZWIK*	OCD: MDD (11%), AD (7.7%), ADHD (3.7%)	OCD: 7%	INH	combined Simon-Go/NoGo	**OCD > HC (commission errors—congruent trials)**	***p*** ** = .026**
			HC: 13.93 (2.05)	27		HC: NR	HC: No			*NS (commission errors—incongruent trials)*	*p* = *.56*

*SCWT* Stroop Color and Word Test, *VCPT* Visual Continuous Performance Test, *BAI* Beck Anxiety Inventory, *BDI* Beck Depressiob Inventory, *RT* reaction time

#The BI effect was smaller in OCD patients (34 ms ± 21 ms) as compared to HC

**Table 4:** Executive functioning in adolescents with post-traumatic stress disorder

Study nr	Paper	Status; Trauma expouse	Mean age (SD)	Sample size	Diagnostic criteria	Psychiatric co-occurrences	Psychotropic medication	EF domain	Task	Results	Statistic
29	*Barrera-Valencia (2017)*	PTSD (current); Physical maltreatment, loss of one/both parents due to armed conflict, living in refugee conditions	PTSD: 12 (2)	23	Interview *MINI-Kid (DSM-IV-TR)*	No	NR	INH	SCWT	**PSTD < HC**	***p*** ** = .015**
			HC:12 (3)	24				SHIFT	WCST	**PTSD < HC (correct)** **PTSD < HC (categories)** **PTSD > HC (perseverative errors)**	***p*** **= .003** ***p*** **= .003** ***p*** **= .002**
30	*Biedermann (2018)*	PTSD (current and lifetime); Childhood sexual abuse (CSA)	PTSD + CSA: 15.95 (1.52)	18	Interview *K-SADS-PL (DSM-IV)*	**PTSD + CSA:** MDD (50%), AD (22.2%), ADHD (5.5%) **PTSD:** MDD (44%), AD (17.6%), ADHD (2.9%) **CSA:** ADHD (12,5%) **HC:** MDD (24%), AD (2.7%), ADHD (5.4%)	NR (sedatives excluded)	SHIFT	TMT-B; WCST	*NS*	*N/A*
			PTSD: 16.03 (1.94)	34				WM	Digit Span	*NS*	*N/A*
			CSA: 15.29 (2.24)	16							
			HC*: 14.66 (1.89)	37							
31	*Park (2014)*	PTSS; Witnessing a deadly accident	HR: 12.42 (0.30)	26	Other *UCLA-CPTSD-RI* > *25*	**HR:** Agoraphobia (7%), GAD (7%) **LR:** NR (but high scores on CDI) **HC:** None	NR	INH	Flanker Task (CAT)	**HR < LR < HC**	**η2 = .161; p < .001**
			LR: 12.45 (0.44)	25				WM	Spatial Working Memory task (CAT)	**HR < HC**	***η2*** ** = ** ***.076; p*** ** = ** ***.036***
			HC: 12.56 (0.28)	30							
32	*Yang (2014)*	PTSD; Earthquake	PTSD: 9–17	34	Interview *MINI-Kid; K-SADS-PL; DSM-IV*	No	NR	INH	SCWT	*NS*	*p* = *.05*
			HC: 9–17	66				SHIFT	TMT-B	*NS*	*p* = *.05*
								WM	Digit Span	*NS*	*p* = *.06*
								INH SHIFT WM	BRIEF-PR	*NS* *NS* *NS*	*p* = .07* *p* = *.20* *p* = *.10*

Abbreviations: *PTSS* post-tramatic stress symptoms, *HR* high-risk group (trauma exposed, high PTSD symptoms (UCLA-PTSD >25)), *LR* low-risk group (trauma exposed, no marked PTSD (UCLA CPTSD-RI <25), depression (CDI <30), or anxiety (STAIC-S <40)), *HC* unexposed healthy controls, *BRIEF-SR *BRIEF self-report, *BRIEF-PR* BRIEF parent-report

*At the 12-month follow-up, PTSD score significantly higher on inhibition than HC (p =.03)

**Table 5:** Summary of studies reporting on significant task - and/or rating-based measures of executive functioning in adolescents

		*INTs* < *HCs*		*HCs* < *INTs*	
		Task-based	Rating-based	Task-based	Rating-based
	*INH*	*S3; S9; S10; * ***S11*** *; S12*	*S6; * ***S11***		
DEP	*WM*	*S1; S2; S5*	*S6*		
	*SHIFT*	*S1; S13*	*S6*		
	*INH*		*S14*	*S15*	
ANX	*WM*		*S14*	*S16*	
	*SHIFT*	*S17*	*S14*		
	*INH*	*S7; S27*	*S21; S22; S25*	*S19; S28*	
OCD	*WM*	***S25***	*S21;* ***S25***		
	*SHIFT*	*S18; S27*	*S21; S25*		
	*INH*	*S29; S31*			
PTSD	*WM*	*S31*			
	*SHIFT*	*S29*			

## Methods

### Search strategy and inclusion criteria

**Fig. 1:**
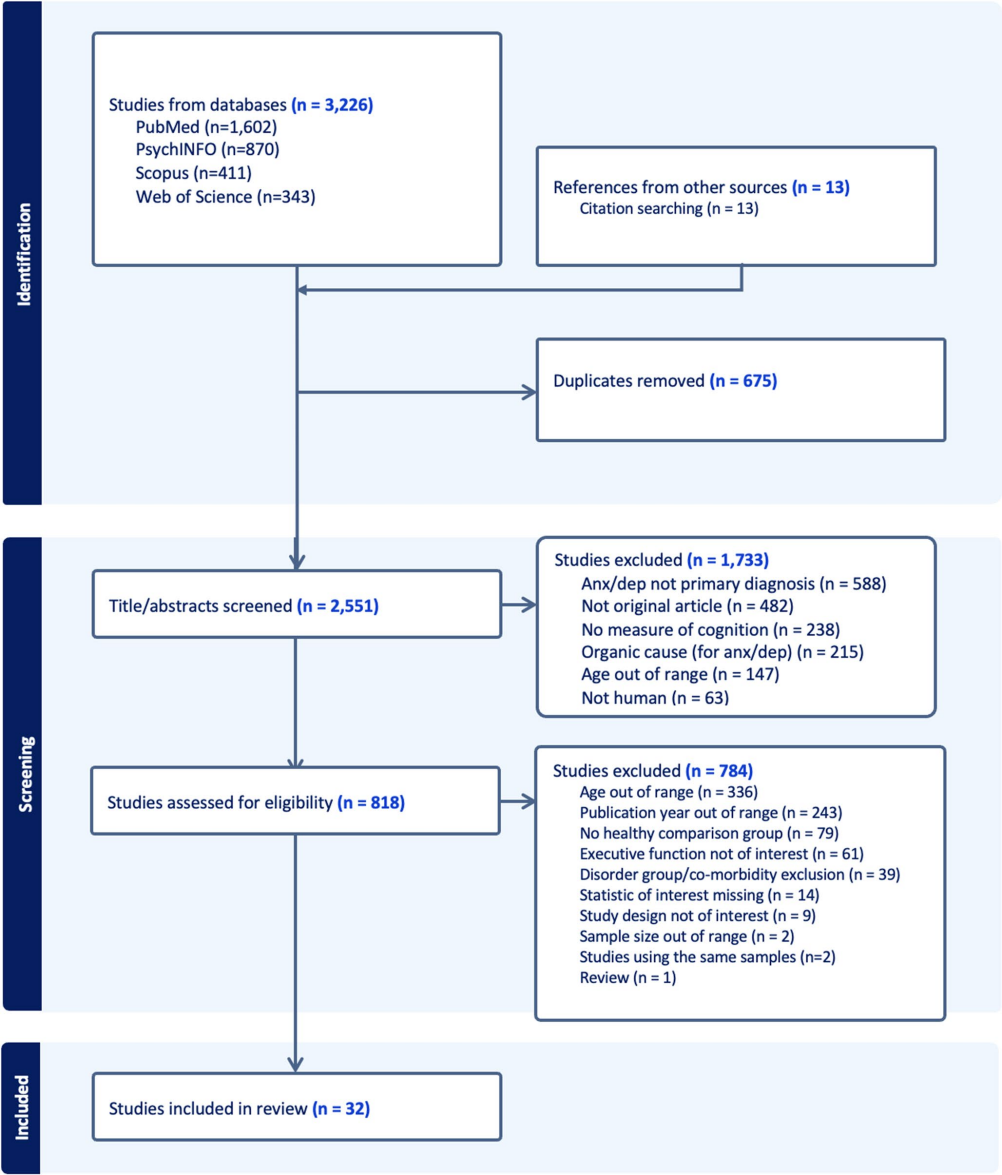
PRISMA flow diagram

We performed a systematic literature search following the Preferred Reporting Items for Systematic Reviews and Meta-Analysis (PRISMA) guidelines [39]. To identify relevant studies, the following databases were searched: Medline (PubMed), Psych-INFO, Scopus and Web of Science. Titles and abstracts were searched with the following search phrase; (Adolescents OR juveniles OR learners OR minors OR puberty OR “high school” OR students OR teens OR teenagers OR youths) AND (“anxiety disorders” OR “depressive disorders” OR “anxiety symptoms” OR “social anxiety” OR “generalised anxiety disorder” OR “generalized anxiety disorder” OR “separation anxiety” OR “panic disorder” OR “phobic disorder” OR “social phobia” OR “depressive symptoms” OR “depressive syndrome” OR “major depressive disorder” OR “dysthymic disorder” OR dysthymia OR “posttraumatic stress disorder” OR “posttraumatic stress symptoms” OR “obsessive-compulsive disorder” OR “obsessive compulsive disorder”) AND (“Executive functioning” OR “executive function” OR “executive dysfunction” OR “set shifting” OR inhibition OR “working memory”). Our search was limited to papers published between the 1 st of January 2014 and the 23rd of May 2023. Figure 1 shows the PRISMA flow diagram of the study selection process.

All abstracts were evaluated using the following inclusion criteria: (a) Original, peer-reviewed articles, written in English and reporting on human participants. (b) Including task-based or self-report measures of cognition. (c) Conducted in teenagers, adolescents, youths, young people. Studies that investigated “adults” were excluded. (d) Depressive and/or anxiety disorders as the primary diagnosis, including obsessive-compulsive disorder (OCD) and post-traumatic stress disorder (PTSD), based on the Diagnostic and Statistical Manual of Mental Disorders (DSM-IV; DSM-5). Following DSM-5 categorization changes, this review considered anxiety disorders, PTSD, OCD, and depressive disorders, as distinct diagnostic entities. Studies were included if they used a structured or semi-structured diagnostic interviews based on DSM-IV/5 or ICD-10 criteria, and/or validated clinical instruments with established cut-off scores derived from DSM or ICD criteria. Furthermore, INTs secondary to a general medical condition (e.g., traumatic brain injury, cancer, HIV, epilepsy, diabetes, heart failure) were excluded. (e) Including a non-clinical control group, free from any mental health diagnosis. Alternatively, norm scores were accepted for EF tasks in INTs.

### Study selection

A total of 2,551 articles were included, after removing duplicates, for the abstract screening. Two reviewers evaluated the abstracts independently and reached a % agreement of 93% (Cohen’s *K* 0.84). Conflicts in decisions were resolved by the third reviewer. A total of 818 articles were included for full-text review. Articles were then excluded based on the following criteria: (a) Papers published before January 2014 to focus on the most recent decade of research and to complement existing reviews of earlier literature [19, 28]. (b) In line with the neurocognitive developmental phase of adolescence [23, 40, 41], we limited the mean age of each group to ≥ 12 and ≤ 17 years. (c) Studies in which clinical cases were not interview-based or not using a self- or parent-report instrument with pre-defined cut-off scores based on the DSM-4/5 or ICD-10. (d) Studies focused exclusively on familial history of any psychopathology. (e) Studies focused on induced anxiety or trait anxiety. (f) To limit confounding, studies including participants with certain psychiatric co-occurrences (e.g., psychosis, bipolar disorder, substance use disorders) or neurodevelopmental disorders (i.e., autism) were excluded. This approach aimed to ensure greater conceptual clarity and a more homogeneous sample of internalizing psychopathology. (g) Emotional (i.e., “hot”) EF tasks were excluded, to avoid performance in EF being confounded with disorder-related alterations in emotional processing [2, 42]. Furthermore, studies that did not test for any of the three core EF domains of interest (inhibition, shifting, WM) - based on Miyake et al.’s [16] unity and diversity model - were excluded. We focus on these core domains because they can be more reliably distinguished from one another compared to more complex EF tasks such as planning and verbal fluency, which draw upon multiple cognitive processes beyond EF, including language processing, visuospatial abilities, and semantic memory [2, 16]. By focusing on these three core domains, we can more precisely identify specific cognitive mechanisms that may be affected in internalizing disorders during adolescence, reducing the potential confounding effects of non-executive processes. We also excluded studies that used novel neuropsychological tasks. However, pre-existing but adapted or modified tasks were accepted and rated as “medium” risk of bias. (h) Clinical trials and intervention studies in which no baseline measure of EF and/or psychopathology was given. (i) Co-morbid medical conditions such as disordered eating (i.e., obesity), cardiovascular issues, neurological disorders (learning disability, seizures disorders, traumatic brain injury), chronic medical illnesses (i.e., diabetes, hypertension) and COVID, to avoid confounding effects. (j) Finally, studies missing critical parameters (e.g., mean/median age) or statistics (e.g., no test statistics for group comparisons), were excluded.

### Data extraction

The following data were extracted from each article: First author, year of publication, sample characteristics (primary psychopathology, sample size, mean age), diagnostic criteria, co-occurrences, psychotropic medication use, EF domain assessed, measures of EF, results, and statistics (Cohen’s *d* and/or *p*-value depending on availability of information). Studies were excluded if corresponding authors were not able to provide relevant information.

Finally, an informal risk of bias assessment was conducted to evaluate each included paper’s choice of diagnostic measure, EF task psychometrics, sample size, clinical configurations and overall risk of bias (see Table 6.).

**Table 6:**
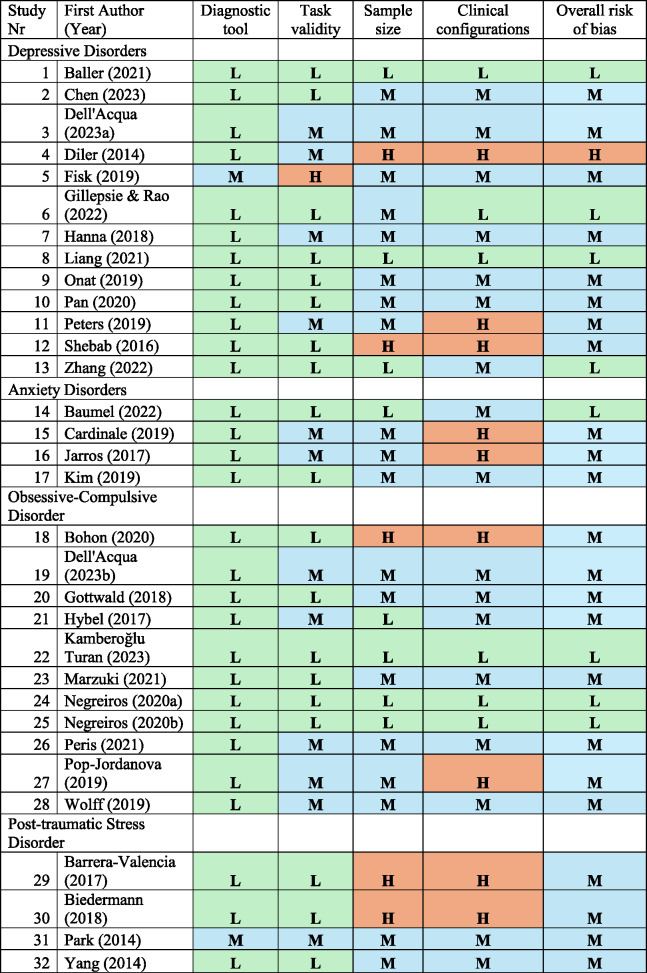
Risk of bias assessment of included studies

L = Low risk of bias

### Synthesis

A narrative synthesis was conducted by grouping studies by disorder type (DD, AD, OCD & PTSD), then further divided into the three core task-based EF domains of interest. Studies reporting on rating-based EF were reviewed separately from task-based EF.

## Results

### Description of included studies

A total of 32 studies investigating EF performance in adolescents with INTs were included in this review. Here, 13 studies focused on DD, with 11 reporting significant differences in one or more core EF domains in cases versus controls (Table 1). Four studies examined AD, all of which found significant differences in at least one core EF domain (Table 2). 13 studies investigated group differences related to OCD, with 8 reporting significant findings in one or more core EF domains (Table 3). Lastly, four studies focused on PTSD, with only two studies reporting significant differences in at least two core EF domains (Table 4). Of the 32 included studies, seven reported on self- or parent-rated EF, of which six found significantly lower ratings. All seven studies used the Behavior Rating Inventory of Executive Function (BRIEF) [22]. Table 5 shows a summary of studies that reported on both task- and rating-based measures of EF. Finally, the quality of studies was assessed, and risk of bias is summarized in Table 6..

### Depressive disorders and task-based core executive functioning domains

#### Task-based inhibition

Nine studies investigated task-based inhibition, with five finding significantly lower performance in depressed adolescents using either the Stroop Color-Word test [43, 44], the Go/No-Go task [45], Flanker Task (FT) [46] or the Rapid Visual Processing Task (RVPT) [47]. Notably, Peters et al. [45] found that only depressed adolescents with a history of childhood trauma, but not DEP, had a lower accuracy on the Go/No-Go task.

Conversely, four studies found no significantly lower performances. Gillespie and Rao [48] found no significant differences on the Color-Word Interference test (CWIT) of the Delis-Kaplan Executive Function System [49], a Stroop-like test. Of note, Gillespie and Rao [48] did not report on psychopathology co-occurrences; whilst Onat et al. [43] and Pan et al. [44] excluded participants with co-occurrences from their MDD sample. While Diler et al. [50] found no differences on the Go/No-Go task, (thus contradicting the findings of Peters et al. [45]); these authors did not account for trauma history, the sample size was relatively small, and high co-occurrence rates were evident. Fisk et al. [51] found no significant differences on the Hayling Sentence Completion task (HSC), a task which exhibits moderate validity and lacks specificity, as it involves the recruitment of language processes. Finally, Hanna et al. [52] found no significant differences between adolescents with MDD and HCs on the FT, contrasting with Dell’Acqua et al. [46]. Of note, 30% of the MDD sample in Hanna et al. [52] were medicated, whilst the sample in Dell’Acqua et al. [46] was unmedicated. Despite these inconsistencies, the findings overall suggest that adolescents with MDD tend to have a lower performance on task-based inhibition.

#### Task-based shifting

In this review, task-based shifting in depressive disorders yielded mixed results. Three out of five studies found no significant differences in adolescents with MDD, either on the Wisconsin Card Sorting Test (WCST) [43, 44, 53] or the Trail Making Test Part B (TMT-B) [44]. In contrast, Zhang et al. [54] reported that adolescents with MDD took longer to complete the TMT-B and made more WCST perseverative errors than controls, with the poorest performance observed in depressed adolescents with non-suicidal self-injury (NSSI). Baller et al. [26] used a data-driven approach to identify cognitive subtypes in adolescents with a lifetime history of a major depressive episode (MDE); and found that, while one subtype had preserved cognitive function, another subtype exhibited lower scores in multiple domains, including shifting as measured by the Penn Conditional Exclusion Test (PCET). Overall, the evidence indicates an absence of major shifting alterations in adolescent depression. Studies suggesting reduced performance may be more closely associated with specific cognitive subtypes [26] or the presence of specific clinical features such as NSSI [54].

#### Task-based working memory

Out of four studies that examined working memory (WM) difficulties in adolescents with DD compared to HCs, three found significant differences on either the N-Back task [26, 53] or the Keep Track Task (KTT) [51]. However, in Chen et al. [53], groups no longer differed after controlling for clinical symptoms. Of note, in Fisk et al. [51] used the Moods and Feelings Questionnaire (MFQ) using clinical cut-offs to define groups (elevated vs. low depressive symptoms), rather than a formal interview-based diagnosis.

In contrast, Liang et al. [55] found no significant differences between adolescents with MDD and HCs, using the Wechsler Memory Scale-III Spatial Span task (WMS-III SS), after controlling for confounding variables. Overall, preliminary evidence suggests a deficit of WM in depressed adolescents, although this effect may be related to symptom severity.

### Anxiety disorders and task-based core executive functioning domains

#### Task-based inhibition

Two studies investigating inhibition in anxious adolescents yielded contrasting results. In their study of 41 adolescents with mixed AD (with either mild (AD-M) or severe anxiety symptoms (AD-S)), and 27 HCs, Jarros et al. [56] reported no significant differences on the Go/NoGo task, between cases and controls. Conversely, Cardinale et al. [57] focused on inhibitory control using an adapted antisaccade task (A-AT) in 27 adolescents with non-specified AD and 19 HCs. While overall accuracy did not differ, the individuals with anxiety exhibited significantly faster latency to correct antisaccades compared to controls (after controlling for age). Higher anxiety symptoms, reported by both parent and child, correlated negatively with saccade latency during correct antisaccade, suggesting *enhanced* inhibitory control. In sum, studies investigating inhibition in anxious adolescence are currently scarce and show mixed findings.

#### Task-based shifting

Two studies examined shifting in adolescents with AD compared to HCs and found contrasting results. Jarros et al. [56] found no significant differences between groups using both the TMT-B and the WCST. Conversely, Kim et al. [58] used the Intra-Extra Dimensional Set Shift (IDED) task on a generalized anxiety disorder (GAD) sample; and found that these participants made significantly more errors on the simple reversal stage compared to HCs, indicating greater cognitive inflexibility. After adjusting for anxiety severity, group differences were no longer significant. Overall, the current findings do not provide sufficient evidence for the presence of lower shifting performance in adolescents with AD.

#### Task-based working memory

Two studies investigated working memory performance of which only one found differences between groups [56]. Kim et al. (2019) found no differences between GAD and HCs on the Spatial Span task (SSP), suggesting preserved visuospatial WM in GAD. Further, Jarros et al. [56] reported that adolescents with mild anxiety performed significantly better than both controls and adolescents with severe anxiety on the Digit Span backward task (a measure of verbal WM), even after controlling for potential confounders.

### OCD and task-based core executive functioning domains

#### Task-based inhibition

Eight studies examined task-based inhibition performance, of which four found significant but contrasting results [52, 59–61]. Hanna et al. [52] found that adolescents with OCD made significantly more errors than HCs on the Flanker Task (FT). Similarly, Pop-Jordanova [61] found that adolescents with OCD had significantly more commission errors (false alarms on NoGo trials) and RT variability on the Visual Continuous Performance test (VCPT) compared to normative data. Further, more interference errors on the SCWT were reported in the OCD sample, compared to controls. By contrast, Wolff et al. [59] reported fewer commission errors on congruent (but not incongruent) No/Go trials in adolescents with OCD, on a combined Simon-Go/NoGo task. Finally, Dell’Acqua [60] found that OCD participants were significantly faster than HCs on error trials, but not in correct trials, on the FT.

Conversely, four studies found no significant differences in task performance between adolescents with OCD and controls on the FT [62, 63] and/or the Stop Signal Task (SST) [62, 64, 65].

Overall, current findings do not suggest major differences in performance, with some preliminary evidence of an inhibition advantage, under certain conditions, in adolescents with OCD.

#### Task-based shifting

Of eight studies examining performance-based shifting in adolescents with OCD, only two found evidence of reduced task performance [61, 66]. Bohon et al. [66] and Pop-Jordanova [61] both reported that on the WCST, adolescents with OCD made more perseverative errors, compared to HCs. This finding was supported by a large effect size in Bohon et al. [66].

Finally, six studies did not find clear differences in shifting between groups, using either the WCST [67], the IDED [58, 62, 64, 65, 68] or the TMT-B [62]; thus suggesting no major alteration in performance in shifting in adolescents with OCD.

#### Task-based working memory

Four studies examined WM performance in adolescents with OCD compared to HCs, of which only one study reported significantly lower performance in the OCD group. Negreiros et al. [64] and Hybel et al. [62] found no differences between groups on the Spatial WM (SWM) task from the Cambridge Neuropsychological Test Automated Battery (CANTAB). On the contrary, Negreiros et al. [65], reported significantly lower scores in OCD-affected adolescents using the same SWM task. Notable differences were the lack of medication use in Negreiros et al.’s [65] control group, as well as higher rates of AD co-occurrences in the case group, compared to Negreiros et al. [64]. Furthermore, Kim et al. [58], using a similar CANTAB Spatial Span task (SSP), found significant group differences, such that the OCD group had shorter span length versus HCs. However, these results were only significant when controlling for anxiety severity.

### PTSD and task-based core executive functioning domains

Four studies examined inhibition, shifting, and WM performance in adolescents with PTSD compared to HCs of which two papers found significant findings [69, 70]. Both studies found significant differences on inhibition tasks between groups. In Park et al. [70] adolescents with marked post-traumatic stress symptoms (PTSS; high-risk) performed more poorly on the FT compared to trauma-exposed adolescents without clinical symptoms (low-risk) and non-trauma exposed controls, even after controlling for co-occurring AD. Furthermore, these authors found significant differences between the high-risk and HCs on a SWM task, but differences were no longer significant after excluding participants with co-occurring agoraphobia and GAD. Of note, no participants met full diagnostic criteria for PTSD in this study and group differences were established using the University of California at Los Angeles post-traumatic stress disorder Reaction Index (UCLA-CPTSD-RI) cut-off score rather than an interview-based diagnosis.

Barrera-Valencia et al. [71] on the other hand found that adolescents with a current diagnosis of PTSD performed significantly less well on both the SCWT (inhibition) and the WCST (shifting). Furthermore, both Biedermann et al. [72] and Yang et al. [73] found no significant differences between groups across EF domains. Importantly, neither studies included a non-trauma-exposed control group, making interpretations difficult. However, Biedermann et al. [72] found significant correlations between severity of childhood sexual abuse and higher reaction times on the TMT-B (shifting), as well as the number of traumatic events and all measures of the WCST (shifting). The above studies all have a “medium” risk of bias (See Table 6.).

### Internalizing disorders and rating-based measures of executive functioning 

Six out of seven included studies reporting on self- or parent-reported EF found significantly lower scores in adolescents with INTs. Of these, two studies used the BRIEF as the only outcome measure of EF [74, 75]. Using the parent-report version of the BRIEF, Kamberoğlu Turan & Turan [75], found that participants with skin picking disorder (SPD) (an OCD-related disorder [76]) had significantly higher (worse) scores than HCs on several EF indexes. However, inhibition was the only core EF that scored significantly higher. Global EF factors of emotional control and planning/organizing were also found to differ significantly between groups. Baumel et al. [74] found that adolescents with GAD scored significantly higher than an age- and sex-matched normative sample, on all BRIEF self-report indexes, including inhibition, shifting and WM. Notably, the prevalence of AD co-occurrence was comparatively high in the AD group.

Furthermore, of five studies using both rating- and task-based measures, three found contradicting findings between modalities [48, 62, 65]. Negreiros [65] found that OCD participants differed significantly from HCs on all core EF captured by the BRIEF parent-report, despite showing specific difficulties only in spatial WM on task-based measures. Of note, these authors also assessed planning abilities, which differed significantly between groups on both task- (d = 0.42) and rating-based (d = 0.89) measures. Similarly, Hybel et al. [62] assessed both task- and parent-report measures of EF and found that while OCD subgroups did not differ from HCs on EF task performance, parent-rated EF behaviors revealed lower scores in all OCD subgroups compared to HCs, except for parent-rated WM in the symmetry/hoarding subgroup. Finally, Gillespie and Rao [48] found that depressed participants scored significantly higher (worse) on the BRIEF self-report Global Executive Composite (GEC), encompassing all 8 EF indexes, compared to HCs, despite the lack of significant differences between groups on the inhibition task. Partial correlations controlling for age and SES showed strong associations between depression scores and the GEC. Of note, the authors did not report on the significance of specific BRIEF indexes.

Two studies found converging results between task- and rating-based EF [45, 73]. Similar to their test-based findings on the Go/NoGo task, Peters et al. [45] found that parents of depressed adolescents with childhood trauma (CT) reported significantly greater inhibitory problems than HCs. Of note, parents of depressed adolescents without CT rated similarly high on the same index but this did not reach statistical significance. Finally, results on the parent-report BRIEF corroborate Yang and colleagues’ [73] findings on their task-based measures EF, with no differences observed between trauma-exposed adolescents with and without PTSD. Of note, this was the only study not reporting statistically significant differences between adolescents with and without INTs, on rating-based EF. However, these authors did not include a non-exposed control group, thus hindering interpretations.

Overall, these findings suggest greater subjective and observed daily behavior problems in inhibition, shifting and WM across DD, AD and OCD.

### Risk of bias assessment

Only 25% of included studies were regarded as overall low risk of bias (*n* = 8 studies), 72% as medium risk of bias (*n* = 23 studies), and 3% as high risk of bias (*n* = 1 study).

Two studies were regarded as medium risk of bias due to concerns in their diagnostics methodology (i.e., validated questionnaires with clinical cut-off scores instead of a formal interview-based diagnosis).

Only 1 study scored high on risk of bias for task validity due to specificity and sensitivity concerns of the HSC task as well as potential contamination by the recruitment of language processes. Studies were considered “medium” (*n* = 12 studies) if they used adapted neuropsychological tasks (i.e., adapted antisaccade) or if the tasks had limited standardization or variable reliability but have been published numerous times in various samples (i.e., Flanker task). Results from these studies should therefore be interpreted with caution.

Five studies scored high on risk of bias due to their samples sizes (i.e., lower than 25 participants in their case group) and 19 were considered medium risk of bias (i.e., between 26 and 50). Only 8 of the included studies had a sample size of cases higher than 50 (i.e., low risk of bias).

Finally, 9 studies scored high on risk of bias for clinical configurations if they did not report on psychiatric co-occurrences or medication use in their sample.

## Discussion

Given inconsistencies in current research on EF difficulties in adolescents with INTs, the aim of this review was to systematically evaluate evidence for disorder-specific neurocognitive markers, specifically in inhibition, shifting and WM. We reviewed 32 studies reporting on the cross-sectional associations between task- and/or rating-based EF and the presence of INTs in adolescents. The results of this systematic review indicate that on task-based measures of EF, only depressed adolescents consistently showed EF difficulties, compared to their healthy counterparts. Although these difficulties were reported across EF domains, they were most robustly present in inhibition and working memory. While findings pertaining to anxiety and related disorders were less consistent, preliminary evidence suggests WM difficulties in adolescent OCD. Rating-based EF, on the other hand, consistently showed a reduced performance across INTs, suggesting global, everyday EF difficulties.

### Domain-specific versus general executive functioning performance

The current findings are in support of the unity and diversity model of EF [1, 16]. The model proposes that while EF share common underlying processes (unity), they also maintain distinct components (diversity). Our findings provide evidence for both aspects: Certain INTs, such as depression and OCD, showed reduced performances across domains, in line with a unity of EF ability. However, performance in some domains were more frequently reduced in certain INTs than in others, suggesting a diversity within its domains. Depression, for example, was found to associate with lower scores in inhibition (*n* = 5 studies) and working memory (*n* = 3 studies) more frequently than in shifting (*n* = 2 studies) tasks. Similarly in OCD, with inhibition (*n* = 2 studies) and shifting (*n* = 2 studies) task performance being slightly more frequently reduced than working memory (*n* = 1 studies). Conversely, improved performance was only reported in inhibition (*n* = 2 studies) in OCD participants.

In this review, the EF difficulties reported to be associated with depression are in line with existing literature in adolescents [19] and adults [25]. Whilst the link between EF and early psychopathology is still poorly understood, a number of explanatory models have started to emerge. One such model is the “impaired disengagement hypothesis” [12, 13], which suggests that executive dysfunction may play a role in the development and persistence of internalising symptoms (i.e., anxiety and depression) by negatively impacting one’s ability to control attention towards emotional information. Depressed individuals may have difficulties disengaging from negative thought patterns, leading to rumination (known for its role in depression [77]). Another model with strong empirical support is the stress generation model [78], positing that stressful life events caused by poor EF (e.g., inability to stay focused, social disinhibition, poor planning abilities) may contribute to internalising symptoms.

While only examined in a limited number of studies (*n* = 3) [56–58] performance-based EF was not found to be significantly reduced in anxious adolescents. Only one study [58] reported significant shifting difficulties in adolescents with GAD compared to both OCD and HCs. This finding aligns with earlier investigations of adolescents with GAD [79] and with INTs more broadly [80], which reported robust associations with cognitive inflexibility. Bloemen et al. [80] posits that shifting difficulties may be a core cognitive deficit in INT, emphasising the link between the inability to flexibly shift attention from negative thoughts (i.e., rumination).

By comparison, two studies with mixed anxiety disorder groups reported an advantage under certain inhibition [57] or WM [56] task conditions. These findings are consistent with a recent meta-analysis of EF in adults with AD [81], reporting that anxious individuals showed increased accuracy, but not speed, in shifting and WM tasks, compared to HCs. One explanation may be that, due to deficient attentional allocation to threat-related stimuli, adolescents with anxiety employ more strategies to compensate for their subjective difficulties while performing in controlled environments [82, 83]. Their task performance may therefore appear similar or improved compared to their non-anxious counterparts. Jarros et al. [56] hypothesize that anxiety may be used functionally by adolescents with mild anxiety, with possible mediation by greater inhibitory control, compared to severely anxious adolescents and controls. Indeed, from an evolutionary perspective, mild anxiety and its associated heightened attention to potential threats may have served an adaptive function in detecting and responding to environmental dangers [84]. This corresponds to work by Cardinale et al. (2019), who reported increased inhibitory control in anxious adolescents, specifically faster latencies in correct antisaccades. This contrasts in part with findings from Majeed’s [81] meta-analysis, in which anxiety disorders in adults were related to slower reaction times but did not show significant differences on accuracy on inhibition tasks.

Of note, Cardinale et al. [57] reported that anxious adolescents performed better than anxious children on inhibitory control measures. Such findings are in line with the important neurodevelopmental period of (late) adolescence, key in the refinement and performance maturation of EF [85], including cognitive control networks [83].

In adolescents with OCD, 4 of 12 studies reported significantly reduced performance on task-based measures of EF (2 INH, 1 WM, 1 SHIFT) [52, 61, 65, 66]. These findings are in line with the existing literature in pediatric OCD samples [86, 87], reporting mixed findings but generally atypical performance across EF; as well as previous meta-analyses of adult OCD [88–90]. To the best of our knowledge, only one meta-analysis has been undertaken on adolescent OCD and neuropsychological performance, reporting small and non-significant effect sizes across all neuropsychological domains - with WM evidencing the smallest effect size [27]. This contrasting finding may be attributable to smaller sample sizes [86]; and/or relatively preserved EF in paediatric OCD [91].

Similar to the literature on AD, two studies found improved performances on measures of inhibitory control in adolescents with OCD [59, 60]. Dell’Acqua [92] reported faster reaction times in error trials, while Wolff et al. [59] reported better accuracy in congruent commissions trials. Wolff et al. [59] suggest this could result from pathological fronto-striatal hyperactivity in OCD leading to intensified ‘braking processes’ mediated by the right inferior frontal gyrus. Interestingly, several studies [52, 60, 63, 66] reporting on neural activation in adolescent OCD, found increased activation irrespective of the direction of differences in performance, suggesting increased efforts.

Finally, only one out of the four studies on PTSD reported significant findings, after adjustment for covariates. Park et al. [70] found that performance in children and adolescents with high PTSS (distinct from PTSD) was significantly reduced on an inhibitory control task. This is in line with a recent meta-analysis reporting that children and adolescents with PTSS show difficulties on inhibition, working memory and shifting tasks, compared to controls [5]. This meta-analysis had wider eligibility criteria than the current systematic review, inclusive of older publication dates, younger age ranges, smaller sample sizes, and higher co-occurrence rates. Of note, the findings from Biedermann et al. [72] and Yang et al. [73] contrast with existing literature on adult PTSD [93, 94] reporting that trauma-exposed individuals with PTSD show lower EF performance than trauma-exposed individuals without PTSD. One possibility is that the exposure to trauma itself, more so in children and adolescents than adults, may impact EF. This is consistent with a meta-analysis of neuropsychological functioning of trauma and PTSD in children, reporting that both trauma-exposed children without PTSD, and trauma-exposed children with PTSD had significantly more difficulties on EF tasks than healthy controls [95]. Thus, further research is warranted in trauma-exposed adolescents with and without PTSD/PTSS, compared to healthy non-exposed control groups.

Our findings reveal important developmental patterns when compared with existing literature on children and adults. In depression, adolescents consistently show lower task performance across all three core EF domains, particularly in inhibition, with less pronounced difficulties in working memory and shifting. This profile mirrors patterns observed in both adults [25] and children [13, 96–98], suggesting depression-related executive difficulties may emerge early in development and persist throughout the lifespan without significant age-related variation.

For anxiety disorders, our review found minimal evidence for lower task-based performance (shifting) in adolescents, which contrasts with more robust difficulties reported in anxious adults [81]. This developmental discontinuity suggests adolescence may represent a unique window where anxiety-related EF temporarily improves. Cardinale et al.‘s [57] observation that anxious adolescents performed better than anxious children on inhibitory control measures supports this pattern. The adolescent neurodevelopmental period appears to offer a protective effect on EF in anxiety, possibly due to the accelerated maturation of prefrontal regions that temporarily compensates for anxiety-related difficulties before declining again in adulthood [99]. In OCD, our findings align with Abramovitch et al. [27], who reported smaller ES for EF difficulties in pediatric compared to adult populations [88–90], suggesting that OCD-related executive dysfunction may progressively worsen over the lifespan rather than remaining stable as seen in depression.

### Task-based versus rating-based executive functioning measures

Having examined findings related to specific EF domains across disorders, we now turn to the important distinction between task-based and rating-based assessment approaches. Our findings align with the current literature, suggesting that rating-based and task-based measures of EF may assess distinct constructs [100]. Studies utilizing latent variable approaches have found only weak-to-moderate correlations between EF task performance and questionnaires in youth and adults [100–102]. Task-based measures assess cognitive performance under structured conditions, and are therefore limited in their ecological validity [103]. Furthermore, because these tasks were initially designed to identify severe neuropsychological deficits, they may lack the sensitivity required to detect subtle changes in EF performance, potentially resulting in smaller effect sizes due to ceiling effects [2]. In contrast, self- and observer-rated measures have demonstrated substantially stronger predictive power for functional indices in psychopathology [104], better sensitivity to EF changes and further show strong concurrent associations with anxiety and depression [4]. Our findings align with this observation [48, 62, 65], indicating the presence of subjective, everyday difficulties that are not detected by behavioral assessments. Alternatively, tasks could potentially capture typical or even improved EF performance under optimized conditions in anxiety and OCD that do not reflect in EF ratings which are based on daily life difficulties. For example, while children and adolescents with OCD may effectively recruit resources during lab-based EF tasks, real-world settings - which tend to include complex emotional and disorder-specific stimuli and draw upon integration of multiple EF domains - may be more challenging [62].

The discrepancy between elevated self-reported EF difficulties and intact task performance across INTs may be explained by two complementary mechanisms suggested by Chevalier et al. [105]. First, the ‘self-absorption paradox’ posits that anxious individuals’ heightened self-awareness makes them more sensitive reporters of subtle EF difficulties. This sensitivity is supported by consistent findings across multiple studies where parent-reports on the BRIEF showed similar elevations [45, 62, 65], suggesting that observed EF difficulties extend beyond self-report biases. Furthermore, the discrepancy between rating and task-based measures was also observed in non-anxious adolescents with depression [48], indicating this phenomenon is not limited to anxiety-specific cognitive biases.

Second, contextual factors likely contribute to this discrepancy. While adolescents with INTs can effectively deploy EF resources in structured laboratory settings, the emotional demands of real-world contexts may compromise their ability to utilize these same skills in daily functioning [102]. Although anxiety disorders may lead to negative cognitive and memory biases that could inflate self-reported difficulties [106, 107], particularly in SAD, this pattern likely reflects both increased metacognitive sensitivity and genuine contextual challenges in applying EF skills in emotionally demanding situations.

Finally, Gillespie and Rao [48] found that BRIEF scores were strongly associated with social adjustment scores, further confirming the ecological validity and importance of this rating-based measure in quantifying real-world difficulties.

### Methodological considerations and limitations

The interpretation of findings across studies is complicated by several methodological factors. First, studies varied considerably in how they handled psychiatric co-occurrences, with some excluding participants with any psychiatric co-occurrence in both case and control groups (*n* = 7 studies), some excluding participants with other psychiatric conditions in the control group only (*n* = 14 studies), while others did not report on psychiatric co-occurrences (*n* = 5 studies). This variability in addressing psychiatric co-occurrences, particularly common in INTs, can contribute independently to EF difficulties, thus confounding the specific associations. The inclusion of participants with Attention Deficit Hyperactivity Disorder (ADHD) in several studies complicates interpretation further, as ADHD is often (but not always) associated with executive dysfunction [108]. Participants with co-occurring ADHD may exhibit EF difficulties due to their ADHD rather than the primary disorder of interest, potentially inflating observed EF difficulties in samples with higher ADHD prevalence [109]. This is further complicated, if participants were medicated for their ADHD (i.e., using stimulants) known to improve cognitive performance [110]. Furthermore, community samples often had higher co-occurrence rates but potentially lower symptom severity than clinical samples - a particularly important consideration for depression and anxiety where co-occurrence is common. Importantly, the presence of psychiatric co-occurrences, particularly in OCD, may reflect real-world clinical presentations. For instance, up to 60% of youth with OCD also meet criteria for an anxiety disorder [83, 111], making the inclusion of comorbid anxiety clinically representative rather than a methodological weakness [85]. Similarly, medication status varied across studies, with some including medicated participants while others used medication-naive samples. Psychotropic medications, commonly prescribed for INTs, are known to potentially improve or decrease cognitive performance depending on medication type [112–114] thus introducing potential biases, and complicating the interpretation of observations. These differences in medication and psychiatric co-occurrence rates might explain contrasting findings between otherwise similar studies [64, 65]. A further challenge lies in task selection and quality (*See* Table 6. *for a Risk of Bias assessment*) as well as the inherent difficulty of measuring pure EF domains. Many commonly used tasks recruit multiple cognitive processes beyond EF. (e.g., WMS-III Spatial Span task engages both visuospatial processing and WM [55] and the use of both inhibition and language processes in the Hayling Sentence Completions Test [51]). This methodological diversity in task design makes it difficult to isolate specific EF difficulties and may contribute to inconsistent findings across studies. Future research would benefit from systematically controlling for medication and co-occurrence status, while using either multiple converging measures for each EF domain or psychometrically strong measures (e.g.,, Stop-Signal Task for inhibition, the N-Back Task for WM, the WCST for shifting) and carefully considering the non-executive processes engaged by each task.

### Clinical implications

Our findings have several potential implications in clinical settings. First, the current review further confirms that different measurement approaches provide complementary - rather than redundant - information. Ratings may better predict real-world functioning, while tasks offer insights into specific cognitive processes [100]. Using both types of measures is recommended to gain a comprehensive assessment of EF abilities and their manifestation in everyday contexts. Researchers and clinicians should be cautious about drawing conclusions across measurement types, as difficulties on EF tasks may not necessarily correspond to EF difficulties in daily life, and vice versa [100, 102].

Further, the relationship between EF difficulties and INTs in adolescents may be more closely associated with symptom severity than diagnostic categories [53, 56, 58, 70]. Studies have found significant EF differences between high-risk and control groups, even when participants did not meet diagnostic criteria (but scored high on symptom measures) [70]. Additionally, symptom severity positively correlates with reduced EF performance, suggesting that scores on specific EF domain might be linked to clinical severity [58, 115]. These findings highlight the importance of considering INTs from both dimensional (symptom severity) and categorical (diagnoses) approaches in research and clinical settings.

Finally, consistent with Fitzgerald et al. [83], interventions targeting the task control circuitry that underlies cognitive control may support brain maturation and symptom improvement in affected youth, offering a promising avenue for early therapeutic strategies.

### Strengths and limitations

This systematic review has several strengths, including its comprehensive examination of both task-based and rating-based EF measures across multiple INT subtypes. The review also adheres to PRISMA guidelines, enhancing its methodological rigor. However, several limitations must be acknowledged. First, the heterogenous quality and small sample sizes across included studies limit the robustness and generalizability of the conclusions. The varying handling of psychiatric co-occurrences and medication use further complicates interpretation of findings. A common limitation of neuropsychological studies in psychiatric samples is the inclusion of medicated participants, especially psychotropics, which are known to affect cognitive function.

Second, the heterogeneity of EF measures used across studies complicates comparisons and prevented a meta-analysis from being conducted. Furthermore, strict adherence to pre-established theoretical concepts [16, 18], prioritizing the specificity of three core EF domains (inhibition, shifting and working memory), inevitably excluded other relevant but more “complex” EF domains, considered as “higher-order” such as planning, verbal fluency, organization and self-regulation [2, 18]. Difficulties in these domains are outside the scope of the present review, but were noted in some reviewed studies [47, 58, 64, 65]. Additionally, methodological limitations regarding psychometric properties of EF tasks used were also present. While the inclusion of studies using validated but adapted or modified tasks (e.g., Combined Simon Go/No-Go, Modified Erikson Flanker Task) allowed broader representation of available literature, these adapted tasks may still possess lower psychometric reliability and results should therefore be interpreted with caution. Inclusion of studies employing clinical symptom cut-offs rather than formal diagnoses may have also impacted diagnostic precision and generalizability of findings. Third, by focusing exclusively on adolescents, this review excluded the important transitional phase from childhood to adolescence. Including studies with younger samples could have offered valuable insights into developmental trajectories of EF. Fourth, limiting the review to studies published in the last decade (2014 onwards) may have left out relevant earlier research. Finally, the focus on cross-sectional studies precludes conclusions about causal relationships between EF difficulties and INTs. Additionally, potential publication bias could have impacted our findings, as research reporting significant differences in EF between groups may have been more likely to be published than studies with null findings.

Despite these limitations, the systematic assessment and transparent reporting of methodological quality within this review allow readers to contextualize findings appropriately and highlight areas for future methodological improvements in the field.

## Conclusion

This systematic review examined EF in adolescents with INTs across 32 studies, specifically focusing on inhibition, shifting, and WM. The findings were mixed across AD, OCD and PTSD and more research is warranted on EF difficulties in these disorders in adolescents. Depressed adolescents however, showed consistent inhibition difficulties, which extends the current body of research. The review further sheds light on the possible compensatory processes at play in anxiety disorders, including OCD. A striking observation are the consistently lower scores shown in rating-based EF measures across INTs, even when task-based measures showed no difficulties, underscoring the importance of using both types of assessments to capture the full spectrum of EF difficulties in clinical populations. Future research should focus on longitudinal studies and use both task- and rating-based measures of EF, to elucidate the developmental trajectories of INTs and EF difficulties and explore potential causal relationships. Overall, this review underscores that executive function difficulties are present across INTs as early as adolescence, emphasizing the need for early comprehensive assessment and tailored interventions that address both cognitive and emotional aspects of these disorders.

## Data Availability

No datasets were generated or analysed during the current study.
